# Irrigation of human prepared root canal – *ex vivo* based computational fluid dynamics analysis

**DOI:** 10.3325/cmj.2012.53.470

**Published:** 2012-10

**Authors:** Damir Šnjarić, Zoran Čarija, Alen Braut, Adelaida Halaji, Maja Kovačević, Davor Kuiš

**Affiliations:** 1 Private dental practice ^“^Dr Mirta Kučan Moraal,” Rijeka, Croatia; 2 Department for Fluid Mechanics and Computational Engineering Technical Faculty, University of Rijeka, Rijeka, Croatia; 3 Department of Restorative Dentistry and Endodontics, University of Rijeka School of Medicine, School of Dentistry, Rijeka, Croatia; 4 Department of Radiology, Medikol Zagreb, Zagreb, Croatia; 5 Private dental practice “Dr sc Maja Kovačević, dr.med.dent.”, Rijeka, Croatia; 6 Department of Oral Medicine and Periodontology, University of Rijeka School of Medicine, School of Dentistry, Rijeka, Croatia

## Abstract

**Aim:**

To analyze the influence of the needle type, insertion depth, and irrigant flow rate on irrigant flow pattern, flow velocity, and apical pressure by *ex-vivo* based endodontic irrigation computational fluid dynamics (CFD) analysis.

**Methods:**

Human upper canine root canal was prepared using rotary files. Contrast fluid was introduced in the root canal and scanned by computed tomography (CT) providing a three-dimensional object that was exported to the computer-assisted design (CAD) software. Two probe points were established in the apical portion of the root canal model for flow velocity and pressure measurement. Three different CAD models of 27G irrigation needles (closed-end side-vented, notched open-end, and bevel open-end) were created and placed at 25, 50, 75, and 95% of the working length (WL). Flow rates of 0.05, 0.1, 0.2, 0.3, and 0.4 mL/s were simulated. A total of 60 irrigation simulations were performed by CFD fluid flow solver.

**Results:**

Closed-end side-vented needle required insertion depth closer to WL, regarding efficient irrigant replacement, compared to open-end irrigation needle types, which besides increased velocity produced increased irrigant apical pressure. For all irrigation needle types and needle insertion depths, the increase of flow rate was followed by an increased irrigant apical pressure.

**Conclusions:**

The human root canal shape obtained by CT is applicable in the CFD analysis of endodontic irrigation. All the analyzed values –irrigant flow pattern, velocity, and pressure – were influenced by irrigation needle type, as well as needle insertion depth and irrigant flow rate.

It is of essential importance that the root canal treatment, besides mechanical cleaning and shaping procedures, includes simultaneous chemical processing (1,2). Chemical processing implies root canal irrigation by various antimicrobial means, which enable the removal of bacteria, necrotic pulp tissue, debris, and smear layer (3). Taking into account the differences between root canal morphology and endodontic files properties, irrigation of root canals is an extremely important addition to mechanical processing (4). The shape of root canals, characterized by numerous irregularities, anastomoses, and curvatures, often does not match the regular geometrical construction and mechanical properties of endodontic files, which results in inefficient and inadequate mechanical treatment of root canals (5). Even though endodontic files, both manual and rotary, are constantly improved (6), up to 50% of root canal surface may remain intact after mechanical processing (7). In these cases, successful endodontic treatment can be provided by additional proper and effective root canal irrigation. The efficiency of irrigation depends on the possibility of mechanical removal of residual necrotic and infected material from the canal by flushing, as well as on chemical activity of the irrigant (4,8).

In terms of chemical activity of the irrigant, different means are used in everyday clinical practice, depending on the desired effect in the root canal (3). The most common and most often used irrigant in clinical practice is sodium hypochlorite (NaOCl), which is applied in concentrations ranging from 0.5 to 5.25% aqueous solution (3). Due to irrigant’s cytotoxicity, it is desirable to completely replace it during endodontic irrigation, while avoiding extrusion into periapical tissue (9).

In terms of physical removal of material from the root canal, irrigation efficiency depends on the possibility of proper application of irrigation system, or proper application depth, irrigation dynamics, irrigant quantity, and irrigant fluid characteristics (4,10-15). In conventional clinical procedure, irrigant is applied during and after root canal cleaning and shaping, by disposable syringes and needles (7,16). Irrigation needles used, such as closed-end, side-vented, or notched open-end needles, vary in diameter and are specifically designed and intended for use in endodontics. It was recommended that the needle should be applied to the working length (WL) of instrumentation, or a millimeter shorter, in the root canal for flushing efficiency and removal of unwanted content from the root canal (17,18). However, recent studies have indicated that efficient irrigant replacement is achieved if the needle is applied to the root canal’s apical third (19-23). However, the effectiveness of flushing in the apical third of the root canal, which is most difficult to access (17), remains questionable (14,24,25).

Computational fluid dynamics (CFD) is a discipline of fluid mechanics that uses numerical methods and algorithms to solve and analyze problems involving fluid flow (gas or liquid) (26). The basis of mathematical modeling that is used for subsequent computer simulations are the Navier-Stokes (NS) equations that define the single-phase fluid flow. Numerous calculations are necessary to simulate the interactions of liquids or gases in contact with surfaces defined as boundary conditions. There are different approaches to solve CFD problems, but they all follow the same basic process (27).

CFD analysis was initially designed for various engineering and industrial purposes. In biomedicine, CFD was introduced in respiratory (28,29) and cardio-vascular (26,30) system research. Recent studies (31,32) confirmed CFD as a powerful analytic tool in the research of endodontic irrigation. The possible shortcomings of previous CFD studies are the strictly geometrical model design and insufficient emphasis on irrigant apical pressure.

The aim of this study was to explore and analyze the influence of the needle type, insertion depth, and irrigant flow rate on irrigant flow pattern, flow velocity, and apical pressure by *ex-vivo* based CFD analysis.

## Materials and methods

Human upper canine tooth, extracted due to periodontal disease at the Clinical Hospital Rijeka, Department of dentistry, was used in this study. The tooth was selected as intact, without caries lesions or fractures, with apical foramen positioned symmetrically. Immediately after extraction, the root surfaces were cleaned, and plaque and tartar removed. The crown was cut to the enamel-cement junction in order to avoid distortions during computed tomography (CT) procedure due to similar radiographic properties of contrast fluid and enamel. The remaining root canal was cleaned and shaped using GT Professional (Dentsply Maillefer, Ballaigues, Switzerland) rotary files to size 40, taper 0.10, at working length of 18 mm. During endodontic instrumentation, the root canal was irrigated with 1% aqueous solution of sodium hypochlorite (33).

After instrumentation, the root canal was conditioned with 20% EDTA (Calcinase, LegeArtis, Dettenhausen, Germany), dried with paper points, and non-ionic contrast fluid (Iopamiro 370, Bracco Industria Chimica s.p.a. Milano, Italy) was introduced in the canal while the root was positioned vertically on a cotton ball and placed in the CT apparatus (Siemens Somathom DRH Sensation 16, Siemens, Munich, Germany). Scan field of view was 160×160 mm, with standard matrix (pixel) format of 512^2^. Scans were realized in volume rendering technique, which resulted in 0.3 mm image resolution.

Dental CT scans were processed and a three-dimensional object in stereo lithography (STL) format representing root and prepared root canal was obtained using Scan IP (Simpleware, Essex, UK) software (34). STL is a file format for describing triangular polygons in three-dimensional space. Root canal lumen was extracted from the initial STL as a separate object and converted to a solid body using computer-assisted design (CAD) software (Catia, Dassault Systemes, Vélizy-Villacoublay, France).

Three different irrigation needle designs were used. Dimensions of an open-ended notched 27G (0.41 mm) irrigation needle (Appli-Vac, Vista Dental Products, Racine, WI, USA) were obtained through measurements performed on digital images taken by stereoscopic microscope, and CAD model was created. This model was used as a template for two virtual needles, one open-ended beveled (injection) needle and a side-vented irrigation needle with a closed tip (based on 30G KerrHawe Irrigation Probe, Sybron Dental Specialties, Orange, CA, USA), both 27G. Virtual needles were designed in order to represent needles of different design but with the same internal and external diameters of 27G needle, which is essential if during simulation a constant flow rate through different needle geometry is desired.

CAD software was further used in proper positioning of needle geometry in the root canal model. Each of the three needle types was placed at 95%, 75%, 50%, and 25% of the WL of the prepared root canal model (0.9, 4.5, 9, and 13.5 mm from the apical tip, respectively) and substracted from root canal solid body.

Objects were exported to commercial CFD software (Star-CCM+^®^, CD-adapco, Melville, NY, USA,). CFD preprocessing included also finite volume mash generation and boundary conditions setting. Four layers of prismatic elements were used to distinguish the boundary layer near the root canal wall, making it 0.02 mm thick. The analysis of the impact of the finite volume cell type (tetrahedral and polyhedral) and the number of finite volume cells was made before the final CFD analysis in order to find the mesh with minimal finite volume cells, which also ensures mesh-independent solution. The number of finite volume cells depends on the needle position and was optimized for each analyzed needle depth. Therefore, the total number of cells in each analysis varied between 1.5-2.5 × 10^6^ of volume polyhedral cells.

This study analyzed the root canal irrigation based on the actual geometry obtained with a CT scan system. The CFD irrigation model used in this study was validated by comparison with numerically determined irrigant penetration by experimental measurement in a previously scanned *ex vivo* prepared root canal. The root canal was filled with 1% NaOCl and irrigated for 10 seconds with the irrigant containing mild concentration of radiographic contrast, which enabled digital radiographic recording and subsequent measurement of irrigant penetration performed using commercial RVG viewer (QuickVision version 3.16.3.8, Owandy, Croissy-Beaubourg, France). Experimental irrigation was performed with the volume flow rates of 0.1 and 0.2 mL/s, using notched open-end needle positioned at 50% and 75% of WL. Experimental measurements on similar examples were also made by Gao et al (31) for the purposes of validation. Therefore, after comparison with experimental data and data obtained from previous study (32), we applied accepted criteria (27,35,36) for quality meshing and selected the appropriate turbulent viscosity model for this type of flow according to numerical results out of four different Reynolds-averaged Navier-Stokes (RANS) models (27,36). The comparison demonstrated that numerically determined irrigant penetration by application of SST k-ω turbulence model was in close agreement to the experimental validation results. The selected model, SST k-ω, consistent with previous CFD endodontic irrigation study (32), was applied to numerical fluid flow analyses of *in vitro* irrigation.

**Figure 1:**
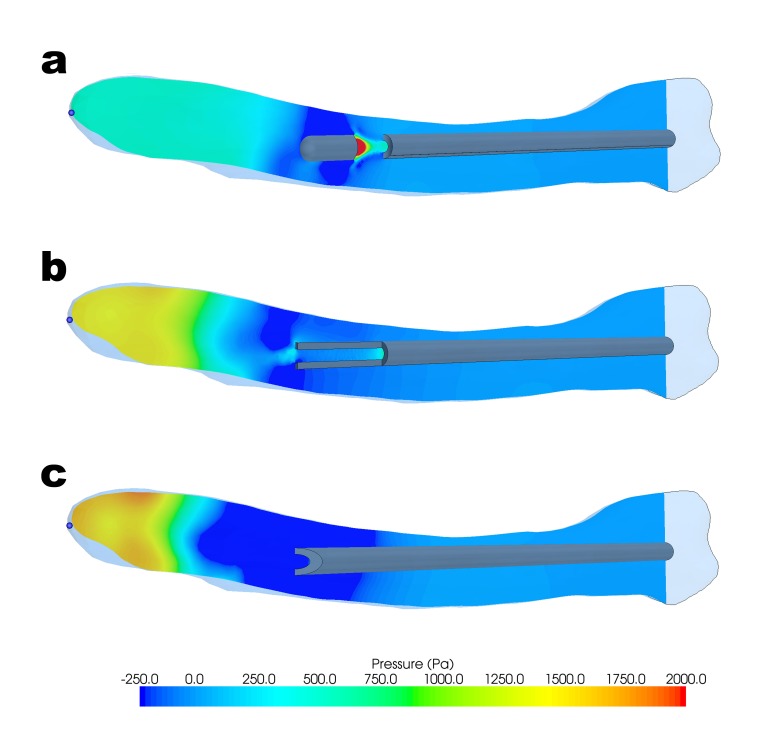
Visualization of the irrigant hydrostatic pressure distribution in the root canal. Three different needles (**A** – closed-end side-vented; **B** – notched open-end; **C** – bevel open-end) positioned at 75% of the WL with 0.3 mL/s irrigant flow rate. Blue dots represent position of pressure probe point (PPP). Images are slightly tilted for enhanced visualization. Contours are color-coded according to hydrostatic pressure magnitude (Pa).

Although CFD analysis provides fluid velocity and pressure data in each mesh cell, two probe points were set in the immediate vicinity of the apical tip of each domain (apical foramen of scanned root canal) and were used to quantitatively measure the pressure and velocity in set points over simulated irrigations using different irrigation needle types, needle insertion depths, and flow rates. The root canal used in this study was designed as “closed,” compared to “open” real root canal with the existing apical foramen (37). At the apical tip of the scanned root canal, pressure probe point (PPP) was established for the measurement of the fluid pressure on the root canal wall (Figure 1). This point corresponds to the position of internal apical foramen and is critical regarding possible irrigant extrusion into periapical tissue.

**Figure 2:**
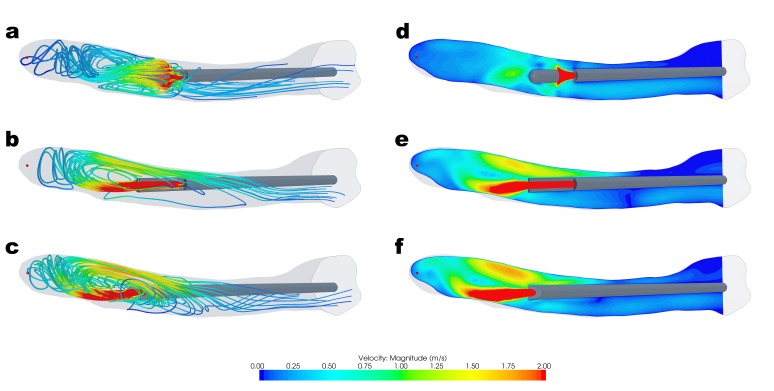
Visualization of the irrigant flow pattern and velocity distribution in the root canal. Three different needles (**A**, **D** – closed-end side-vented; **B**, **E** – notched open-end; **C**, **F** – bevel open-end) positioned at 75% of the WL with 0.3 mL/s irrigant flow rate. Red dots represent position of velocity probe point (VPP). Images are slightly tilted for enhanced visualization. Stream-lines (**A**-**C**) indicating the route of mass-less particles released from the needle aperture and corresponding contours (**D**-**F**) are color-coded according to velocity magnitude (m/s).

The velocity probe point (VPP) was set at a distance of 0.2 mm from the root canal wall (0.2 mm distance from PPP) (Figure 2), well outside the boundary layer, in order to minimize the fluid flow interference from the solid wall, where flow velocity approaches zero. The boundary layer thickness is the distance across a boundary layer from the wall to the point where the flow velocity has reached the “free stream” velocity. The exact boundary layer thickness is difficult to determine in cases of complex geometries, such as this one.

Boundary conditions settings in this study simulated intended clinical conditions. Root canal walls were set as impermeable, resembling dentine walls of the root canal. The root canal wall was defined as no slip wall, which ensures zero velocity at solid walls. Gravity factor was directed at 90° with respect to the upper canine root canal axis when the patient is seated in the dental chair for endodontic treatment.

The irrigation fluid was defined as a 1% solution of sodium-hypochlorite (NaOCl) in water so the computational properties of the fluid mixture were derived from individual properties of both pure liquids using a volume-weighted approach. The resulting irrigant properties used in this study were assumed to be an incompressible, homogeneous Newtonian fluid with a constant viscosity of 0.889·10^-3^ kg/ms and density of 997.6 kg/m^3^.

For the outlet section of the needle, mass flow inlet boundary condition was selected. Irrigant mass flow rate was determined from the volume flow rate, which was set to five, clinically realistic, different values for each needle type and position. Volume flow rates were set to 0.05, 0.1, 0.2, 0.3, and 0.4 mL/s, and corresponded to clinically reproducible values.

Turbulent quantities (k and ω) at the inlet were estimated using prescribed turbulence intensity and hydraulic diameter. Turbulence intensity (I), which is defined as ratio of the root-mean-square of the velocity fluctuation (*u*’) to the mean flow velocity (*u_avg_*),**is totally dependent on the upstream history of the flow. For the present study, turbulence intensity was estimated from the formula derived from an empirical correlation for pipe flows:


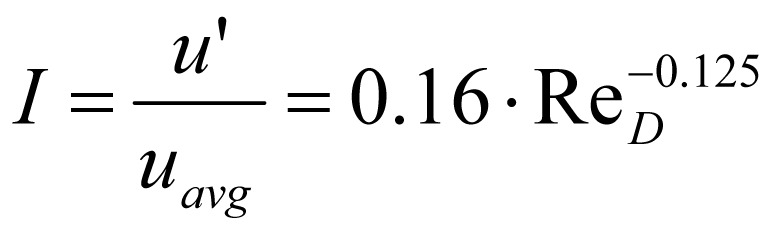


According to this formula, turbulence intensity was set to 5-8% depending on the analyzed Reynolds number (analyzed needle mass flow rate). Hydraulic diameter value was set to equal the actual needle internal diameter.

A pressure outlet boundary condition was imposed at the root canal orifice to allow irrigant outflow from the domain. Atmospheric pressure was assumed at the outlet.

Steady-state conditions were deemed sufficient for the CFD analysis of root canal irrigation, based on point probe analyses of velocity and pressure variations in an unsteady CFD analysis. Our pre-experimental calculations showed that pressure and velocity remained unchanged after just 0.07 s, which is about 1/140 of the total time elapsed during irrigation or only 0.7%, thus justifying the computationally cheaper steady-state method.

Fluid flow simulations were performed using the commercial fluid flow solver Star-CCM+^®^, where a set of equations is solved using standard finite volume techniques. Equations are integrated over individual computational cells and, in case of unsteady simulations, over a finite time increment. The second-order upwind scheme was used to calculate convective flux on the boundary surfaces of control volumes. This second order scheme is the least sensitive to mesh structure and quality. The SIMPLE algorithm (27), which uses a relationship between velocity and pressure corrections, was used to ensure mass conservation and to obtain the pressure field.

CFD analyses were performed with 60 simulations (3 needle types at 4 positions with 5 flow rates). Computations were carried out on HP BladeSystem (Hewlett-Packard Co., Palo Alto, CA, USA) cluster running Red Hat Linux. BladeSystem cluster consists of 10 nodes interconnected with high throughput, low latency Infiniband network. Each node contains two 4-core Xeon 2.66GHz processors with 8GB RAM memory. A total of 80 cores were available for solving time-averaged, three-dimensional RANS equations of incompressible fluid, by an iterative algorithm solver until convergence was reached. The simulations were performed and analyzed in 2010 and the beginning of 2011.

## Results

### Needle application to the depth of 25% of the WLP

*Side-vented closed-end needle*. Fluid movement was intense at the needle aperture with visible lateral jet flow pattern, which corresponded to the position of the needle aperture, and suddenly turned in the apical direction. Moderate fluid movement was visible around the tip of the needle in the apical direction and slightly increased with the increase of flow rate, but it did not exceed the coronal third of the root canal except at the flow rate of 0.3 and 0.4 mL/s, when it reached the middle third of the canal. There was no fluid movement in the apical third of the root canal (Table 1).

**Table 1:** Irrigant fluid velocity measured at velocity probe point (m/s)*

	Irrigation needle position							
Irrigation needle type	at 25% of WL		at 50% of WL		at 75% of WL		at 95% of WL	
	volume flow rate (mL/s)	fluid velocity (m/s)	volume flow rate (mL/s)	fluid velocity (m/s)	volume flow rate (mL/s)	fluid velocity (m/s)	volume flow rate (mL/s)	fluid velocity (m/s ^1^)
Closed-end side-vented	0.05	3.88E-10	0.05	1.93E-10	0.05	2.37E-07	0.05	4.13E-03
	0.1	3.32E-10	0.1	5.48E-08	0.1	1.98E-06	0.1	3.45E-02
	0.2	4.38E-10	0.2	5.68E-07	0.2	1.29E-04	0.2	1.42E-01
	0.3	1.29E-07	0.3	1.30E-06	0.3	7.62E-02	0.3	2.14E-01
	0.4	2.57E-06	0.4	2.57E-06	0.4	2.03E-01	0.4	2.48E-01
Notched open-end	0.05	8.78E-12	0.05	8.58E-11	0.05	1.05E-04	0.05	9.68E-02
	0.1	9.33E-12	0.1	6.82E-10	0.1	9.32E-03	0.1	2.67E-01
	0.2	9.15E-11	0.2	1.78E-07	0.2	7.16E-02	0.2	6.70E-01
	0.3	8.91E-11	0.3	9.64E-05	0.3	1.78E-01	0.3	1.10E+00
	0.4	1.86E-10	0.4	6.40E-04	0.4	3.03E-01	0.4	1.59E+00
Bevel open-end	0.05	6.47E-12	0.05	2.22E-10	0.05	7.50E-04	0.05	2.48E-01
	0.1	3.79E-11	0.1	5.69E-09	0.1	1.46E-02	0.1	5.06E-01
	0.2	7.74E-08	0.2	2.91E-06	0.2	6.50E-02	0.2	1.06E+00
	0.,3	1.06E-06	0.3	6.44E-05	0.3	2.88E-01	0.3	1.61E+00
	0.4	8.18E-08	0.4	1.35E-03	0.4	3.39E-01	0.4	2.19E+00

*Abbreviations: WL – working length, E – exponent value (10^×^).

*Notched open-end needle*. There was a notable output stream directed apically and slightly tilted, corresponding to the position of the needle aperture. Vortex flow patterns were visible in the apical and lateral direction from the needle aperture. Fluid movement was reduced to the coronal third of the root canal, but at the flow rate of 0.2 mL/s the movement entered the middle third, increasing to the flow rate of 0.4 mL/s, at which the movement was evident throughout the middle third of the root canal. Although flow pattern of notched open-end needle, compared to side-vented closed-end needle, showed increased fluid flow penetration, there was no fluid movement in the apical third of the root canal (Table 1).

*Bevel open-end (injection) needle*. The fluid flow pattern was very similar to the flow pattern around the notched open-end needle. The difference was somewhat greater speed and more vortex-shaped fluid movement. Changes in the fluid pattern in relation to the increasing flow rate were also similar, except that the fluid movement in the middle third of the root canal was evident already at flow rate of 0.1 mL/s. There was no fluid movement in the apical third of the canal (Table 1).

### Needle application to the depth of 50% of the WL

*Side-vented closed-end needle*. Fluid flow pattern was identical as at the depth of 25% of the WL, but due to the position of the needle fluid movement was now reduced to the middle and coronal third of the root canal, except at the 0.3 and 0.4 mL/s flow rate, when moderate fluid movement in the initial part of the apical third was apparent.

*Notched open-end needle*. Fluid flow pattern was identical as at the depth of 25% of the WL. Even though at the flow rate of 0.2 mL/s fluid movement was evident in the apical third of the root canal, it remained at the same level as in side-vented closed-end needle flow pattern, regardless of the increasing flow rate (Table 1).

*Bevel open-end (injection) needle*. The only difference from the findings obtained using notched open-end needle was higher fluid inlet velocity for each flow rate analyzed.

### Needle application to the depth of 75% of the WL

*Side-vented closed-end needle*. Fluid movement was apparent throughout the entire length of the root canal at the flow rate of 0.3 (Figure 2D) and 0.4 mL/s, where fluid flow pattern indicated potentially efficient irrigant replacement (Figure 2A).

*Notched open-end needle*. Fluid movement was apparent throughout the entire length of the prepared root canal at the flow rate of 0.2-0.4 mL/s (Figure 2E), where fluid flow pattern also indicated potentially efficient irrigant replacement (Figure 2b).

*Bevel open-end (injection) needle*. Fluid flow pattern was identical as for open-end notched needle at each flow rate (Figure 2C). Fluid flow pattern also indicated potentially efficient irrigant replacement at the flow rates of 0.1-0.4 mL/s (Figure 2F, Table 1)

### Needle application to the depth of 95% of the WL

*Side-vented closed-end needle*. Fluid movement was apparent throughout the entire length of the root canal at the flow rate of 0.1-0.4 mL/s. Flow patterns at 0.3 and 0.4 mL/s flow rate were identical.

*Notched open-end needle*. Fluid movement was apparent throughout the entire length of the prepared root canal at all the analyzed flow rates. Fluid flow pattern indicated potentially efficient root canal flushing at the flow rates of 0.1-0.4 mL/s.

*Bevel open-end (injection) needle*. Fluid movement was apparent throughout the entire length of the root canal. Flow pattern, indicating potentially efficient prepared root canal flushing, was apparent at all the analyzed flow rate values.

For all irrigation needle types and application depths, increase in irrigant flow rate induced an increase in irrigant apical pressure measured at PPP (Table 2).

**Table 2:** Irrigant fluid pressure measured at pressure probe point (Pa)*

	Irrigation needle position							
Irrigation needle type	at 25% of WL		at 50% of WL		at 75% of WL		at 95% of WL	
	volume flow rate (mL/s)	fluid pressure (Pa)	volume flow rate (mL/s)	fluid pressure (Pa)	volume flow rate (mL/s)	fluid pressure (Pa)	volume flow rate (mL/s)	fluid pressure (Pa)
Closed-end side-vented	0.05	11.74	0.05	17.48	0.05	22.28	0.05	30.65
	0.1	50.79	0.1	67.74	0.1	82.82	0.1	120.55
	0.2	109.38	0.2	232.21	0.2	301.55	0.2	448.17
	0.3	403.69	0.3	466.70	0.3	604.66	0.3	952.00
	0.4	705.51	0.4	842.25	0.4	1062.53	0.4	1611.45
Notched open-end	0.05	24.19	0.05	31.54	0.05	42.64	0.05	80.79
	0.1	101.42	0.1	121.87	0.1	162.15	0.1	333.87
	0.2	427.56	0.2	496.97	0.2	683.12	0.2	1520.38
	0.3	952.31	0.3	977.34	0.3	1546.14	0.3	3639.58^†^
	0.4	1674.48	0.4	1813.43	0.4	2739.14^†^	0.4	6807.80^‡^
Bevel open-end	0.05	32.48	0.05	39.73	0.05	54.62	0.05	131.62
	0.1	127.91	0.1	144.54	0.1	206.70	0.1	514.11
	0.2	496.85	0.2	541.03	0.2	828.51	0.2	2124.26
	0.3	1037.17	0.3	1127.04	0.3	1753.52	0.3	4775.32^‡^
	0.4	1782.28	0.4	1980.45	0.4	3208.46^†^	0.4	8309.22^‡^

*Abbreviations: WL – working length.

†Pressure values enter physiological interstitial tissue pressure (ISTP) range 2666-3999 Pa.

‡Pressure values exceed the physiological ISTP range.

## Discussion

All the analyzed values; irrigant flow pattern, velocity, and pressure were influenced by application of different irrigation needle types, as well as needle insertion depth and irrigant flow rate. Irrigation needle insertion closer to WL in the analyzed canal model resulted in increased irrigant apical pressure and velocity.

Although CFD was recognized as a powerful numerical tool in the analysis of endodontic irrigation (31,32), a further development of experimental model is needed due to the complexity of fluid phenomena occurring during the irrigation process. Credible results of clinical relevance are obtained by a realistic simulation of the factors that affect the irrigation dynamics, therefore CFD analysis in the present study was additionally enhanced by CT mapping and application of the human root canal morphology.

According to irrigant flow pattern analysis, closed-end side-vented needle required insertion depth closer to WL regarding irrigant replacement efficiency, compared to open-end irrigation needles. Open-end irrigation needle types showed increased apical pressure and velocity compared to closed-end side-vented needle, which is consistent with a previous CFD study (23).

For all analyzed irrigation needle types and needle insertion depths, the increase in the flow rate was followed by increased apical irrigant pressure. Simulated irrigation flow rate values in this study are clinically realistic and are in agreement with previous CFD irrigation studies (30,31). The flow rate value of 0.1 mL/s is consistent with the flow rate used in CFD irrigation research (0.1 g/s) by Shen et al (23), while the flow rate value of 0.2 mL/s is consistent with the flow rate value obtained by Hsieh et al and Sedgley et al (21,22). The range of irrigant flow rate (0.05-0.4 mL/s) in this study was defined for quantified, enhanced analysis of correlation between the resulting values of flow rate, apical irrigant velocity, and apical irrigant pressure.

Apical pressure in this study was lower (Table 2) than in the study by Shen et al (23) for the same needle diameter, type, flow rate, and similar needle distance from the WL, though in both studies #40 rotary file was used for final preparation. This difference can be explained by greater taper of the rotary file used in this study, which resulted in a more voluminous root canal, confirming the results of previous studies that taper size (20) or root canal enlargement (21) affected irrigation dynamics.

CFD analysis provides vivid display of endodontic irrigation dynamics, hence virtual irrigation needles in this study were also positioned at 25 and 50% of WL (9 and 13.5 mm from the apical tip), contrary to the clinical recommendations. Despite the controversy related to these needle positions, the intention was to provide CFD perspective of irrigation at these needle placements. Irrigation needle insertion in the cervical (25% of WL) and middle portion (50% of WL) of the root canal showed inefficient irrigant replacement for all analyzed needle types and flow rate values. More importantly, the probe point data showed that, although ineffective regarding irrigant replacement, short needle insertion still enabled substantial apical irrigant pressure increase, consistently with the increase in flow rate. Ineffective irrigant replacement for short needle insertion is supported by the results of the endodontic irrigation study (21), where irrigation needle was positioned 9 mm from WL.

The probe points, PPP and VPP, were positioned in the proximity of the apical tip, providing fluid pressure and velocity data that are essential for irrigation efficiency assessment in the apical portion of the root canal. PPP corresponds to the position of internal apical foramen and is critical regarding possible irrigant extrusion into periapical tissue, while VPP provides measurement data relating to the assessment of irrigant replacement. If other irrigation needle types or protocols are analyzed in the future, probe point concept could provide the opportunity for numerous irrigation regimen simulations and comprehensive presentation of study results.

When the irrigation needle is applied to the required root canal depth, there is a possibility of irrigant periapical extruding, due to increased apical pressure, causing complications that result from cytotoxic effects of irrigants (particularly sodium hypochlorite) (14,17,38-40). The question is whether the controlled application prevents the extruding and what the critical flow rate and pressure of irrigant is.

Guyton (41) measured interstitial tissue pressure (ISTP) and concluded that in physiological conditions it ranged between 2666 and 3999 Pa (20-30 mmHg), mean value 3333 Pa (25 mmHg, MISTP). MISTP may represent the threshold above which periapical extruding is possible. Taking further into account that pathological conditions can reduce ISTP to 1333 Pa (10 mmHg), the apical pressure control during irrigation gains in importance. This assumption is supported by Salzgeber et al (18), who reported increased irrigant penetration and apical extrusion in cases of root canals containing necrotic pulp tissue versus cases of root canals with vital pulp tissue.

Though interstitial tissue pressure of vital and necrotic pulp was studied (42), there is lack of studies on periapical tissue pressure. Periapical ISTP, normal and pathological, may be essential for further CFD analysis development and assessment of possible periapical irrigant extrusion. Irrigant pressure thresholds are a complex and vague issue at this level of endodontic irrigation studies. Similar is the complexity of thresholds regarding irrigant velocity values that could ensure efficient flushing action of debris in the apical third of the root canal, critical for successful endodontic treatment, so we encourage further scientific studies and discussion on the issue.

In conclusion, the human canine root canal shape obtained by CT used in this study is applicable for defining fluid flow domain for CFD analysis of endodontic irrigation of large and straight root canals using commercial fluid flow solver.
